# Comprehensive investigation of gene mutations in canine large cell gastrointestinal lymphoma

**DOI:** 10.3389/fvets.2025.1535446

**Published:** 2025-02-19

**Authors:** Naoki Matsumura, Takumi Tsuruta, Yuko Goto-Koshino, Keijiro Mizukami, Tomomi Aoi, Ryoko Yamada, Yuki Matsumoto, Itsuma Nagao, Megumi Sakamoto, Taisuke Nakagawa, Ray Fukuoka, Aki Ohmi, James K. Chambers, Kazuyuki Uchida, Yukihide Momozawa, Hirotaka Tomiyasu

**Affiliations:** ^1^Laboratory of Veterinary Internal Medicine, Department of Veterinary Medical Sciences, Graduate School of Agricultural and Life Sciences, The University of Tokyo, Bunkyo-ku, Tokyo, Japan; ^2^Veterinary Medical Center, Graduate School of Agricultural and Life Sciences, The University of Tokyo, Bunkyo-ku, Tokyo, Japan; ^3^Laboratory for Genotyping Development, RIKEN Center for Integrative Medical Sciences, Kanagawa, Japan; ^4^Anicom Specialty Medical Institute Inc., Shinjuku-ku, Tokyo, Japan; ^5^Laboratory of Veterinary Pathology, Department of Veterinary Medical Sciences, Graduate School of Agricultural and Life Sciences, The University of Tokyo, Bunkyo-ku, Tokyo, Japan

**Keywords:** dog, targeted next-generation sequencing, T-cell lymphoma, whole exome sequencing, *ZDBF2*

## Abstract

Large cell gastrointestinal lymphoma (LCGIL) is the most common extranodal lymphoma in dogs, but its molecular biological backgrounds have not been clarified. In this study, we comprehensively investigated the gene mutations in LCGIL. Whole exome sequencing analysis using four dogs with LCGIL showed mutations in *NACC1* gene in two dogs. Further, the six genes known to be mutated in human intestinal T-cell lymphoma, *ASXL3, SOCS3, PRDM1, FYN, TET2,* and *ZDBF2,* were found to be mutated in one dog. Then, targeted next-generation sequencing analysis was performed to validate these results using additional 31 dogs with LCGIL. As a result, the mutation in *ZDBF2* genes were identified in all samples, but the same mutation was ubiquitously observed in all peripheral blood samples. As for the remaining genes, the mutations were not observed in any dogs. The targeted next-generation analysis of whole exon regions of *ZDBF2* revealed the other mutations in additional three dogs. In the present study, some mutations in genes related to human intestinal T-cell lymphoma were identified, but common gene mutations were not found among most cases. These results implied the heterogeneity of molecular pathophysiology of canine LCGIL. Further studies are needed to comprehensively analyze genomic and non-genomic molecular aberrations in each canine LCGIL case.

## 1 Introduction

Gastrointestinal lymphoma is the most common extranodal lymphoma in dogs, accounting for 5–7% of all lymphomas (1). Affected dogs exhibit non-specific digestive symptoms such as weight loss, diarrhea, vomiting, and anorexia. Most canine gastrointestinal lymphoma is of T-cell origin and commonly affects small intestine (2). Gastrointestinal lymphomas are further classified into large cell and small cell lymphomas based on cell size, and cases with large cell gastrointestinal lymphoma (LCGIL) are usually treated with multi-drug combination chemotherapy including CHOP regimen or lomustine, resulting in a median survival of 62–72 days (3–5).

Several studies have comprehensively searched for gene mutations in canine lymphomas. Whole exome sequencing (WES) analysis revealed common mutations in *TRAF3, MAP3K14, FBXW7,* and *POT1* genes in nodal B-cell lymphomas of Golden Retriever and Cocker Spaniel, which are predisposed breeds of lymphoma (6). Another study using dogs with diffuse large B-cell lymphoma (DLBCL), which is the most common type in dogs, reported common mutations in 8 genes in more than 15% of the cases: *TRAF3, SETD2, POT1, TP53, MYC, FBXW7, DDX3X*, and *TBL1XR128* genes (7). On the other hand, little overlap in the mutation patterns was observed in the T-cell lymphoma of Golden Retriever and Boxer (6). However, these studies did not focus on other specific lymphoma types including LCGIL.

In human T-cell lymphoma, somatic gain-of-function mutations in Janus kinase/signal transducer and activator of transcription (JAK–STAT) pathway are known to be characteristic and suggested to be the main drivers in the malignant transformation. The *JAK1* and *STAT3* gene mutations are observed in 90% of Enteropathy-associated T-cell lymphoma (EATL) patients and 80% of refractory celiac disease type II (RCD2) patients (8, 9). Also, the studies on monomorphic epitheliotropic intestinal T-cell lymphoma (MEITL) have reported frequent gain-of-function mutations in *JAK3* and *STAT5B* genes (10, 11). In addition, a study comparing gene mutations between canine and human tumors showed that canine DLBCL and T-cell lymphomas share the similarities in gene and pathway alterations, such as those in Wnt and p53 signaling pathways, with respective lymphoma types in humans (12).

Based on these findings, we recently investigated the mutations in *STAT3, STAT5B,* and *JAK1* genes in 31 canine cases with LCGIL, but only two and one dog had the mutations in *STAT3* and *JAK1* genes, respectively (13). Therefore, the present study aimed to find mutations in novel genes in canine LCGIL by comprehensive investigations of genetic mutations in canine LCGIL using WES and targeted next-generation sequencing analysis (targeted NGS).

## 2 Materials and methods

### 2.1 Case selections

This study included 35 dogs referred to the Veterinary Medical Center of the University of Tokyo (VMC-UT) and diagnosed with LCGIL. All of the dogs originated from Japan. The diagnosis of large cell lymphoma was made based on histopathological examinations using endoscopically or surgically obtained tissues or cytological examinations using samples obtained by fine-needle aspiration. PCR for antigen receptor gene rearrangements (PARR) was conducted using a part of DNA derived from tumors as previously described to evaluate clonal rearrangements of the *T-cell receptor γ* (*TCRγ*) and the *immunoglobulin heavy chain* (*IgH*) genes in all cases (14). Peripheral blood-derived gDNAs (genomic DNA) were available in 21 of the 35 cases and analyzed as normal controls. The signalment and sample information of each case are shown in Supplementary Table S1.

### 2.2 WES analysis

WES analysis was conducted using 4 dogs diagnosed with LCGIL (Dog 1–4). gDNAs (500 ng) obtained from the tumor site sample were mechanically sheared to fragments of approximately 150 bp (Covaris S220, Woburn, MA, USA). Fragment sizes were verified for quality control using Agilent 2,100 Bioanalyzer (Agilent, Santa Clara, CA, USA). SureSelect XT Canine All Exon V2 kit (Agilent, Santa Clara, CA, USA) and AMPure XP beads (Beckman Coulter, CA, USA) were used to prepare libraries for sequence analysis. The library was sequenced using a paired-end 2 × 100 bp read protocol on the NextSeq500 instrument (Illumina, San Diego, CA, USA). Signal captures were converted into FASTQ files using bcl2fastq (v2.18.0.12) and trimmed with trimgalore (v0.6.5) and Cutadapt (v1.15). The alignment of processed reads to a canine reference genome (CanFam 3.1, GenBank assembly accession: GCA_000002285.2), local realignment, and variants calling in each sample were performed using DRAGEN software (v 07.021.572.3.6.3) (Illumina, San Diego, CA, USA) with the standard protocol, and mutations were annotated using SnpEff (v5.0). The information on canine genetic variation, single nucleotide polymorphisms, and insertions and deletions was available from the Ensembl variation database (CanFam3.1, dog release 101). Somatic mutations were called using VarScan2. Somatic mutations whose variant allele frequencies >0.2 and somatic *p* value <0.05 were regarded as somatic mutations. Extracted mutations in WES were filtered by the following criteria: (1) mutations that were categorized into HIGH and MODERATE by SnpEff, (2) mutations with total read depths of more than 15 and variant read depths of more than 5 in the tumor genome, (3) mutations with total read depths of more than 15 and variant allele frequency of less than 0.03 in the peripheral blood genome. Datasets obtained through WES analyses are available at the DDBJ Sequenced Read Archive repository with accession number PRJDB15563.

### 2.3 Targeted NGS analysis

The mutations that were extracted based on the results of WES were validated by targeted NGS with the samples used for WES and additional samples from 31 dogs with LCGIL (Dog 5–35, Supplementary Table S1) as a previous study (15). The sequencing libraries for targeted NGS were prepared from each gDNA sample by two-step multiplex PCR; 1st PCR to amplify the target regions using Platinum™ Multiplex PCR Master Mix (Applied Biosystem, CA, USA), and 2nd PCR to add index sequences, which were used to identify individuals, and sequences that were used to bind the flow cell using KOD One PCR Master Mix (TOYOBO, Osaka, Japan). The library was purified using AMPure XP beads (Beckman Coulter, CA, USA). After assessing the quantity and quality using Bioanalyzer (Agilent, Santa Clara, CA, USA) and KAPA Library Quantification Kit (Nippon Genetics, Tokyo, Japan), the library was sequenced on the MiSeq instrument (Illumina, San Diego, CA, USA). FASTQ files were trimmed with Cutadapt (v2.10). The processed reads were mapped to CanFam 3.1 using BWA (v0.7.17) and sorted using Samtools (v1.10). Variants calling in each sample were performed using the Mutect2 tool in GATK (v4.2.6.1). Primer sequences used for targeted NGS are shown in Supplementary Table S2. Variants were evaluated as mutations if the variant allele frequencies were more than 0.1.

In addition, we investigated the mutations of the *ZDBF2* gene throughout the whole coding region by targeted NGS analysis that covered all exons using the samples from 31 LCGIL dogs (Dog 5–35). Targeted NGS analysis was performed according to the same method described above using ROS_Cfam_1.0 as a reference genome. Primer sequences are shown in Supplementary Table S3. Variants were evaluated as mutations by the following criteria: (1) mutations with the variant allele frequencies of more than 0.1 and the total read depths of more than 100 in the tumor sample, (2) mutations with the variant allele frequency of less than 0.1 and the total read depths of more than 100 in all peripheral blood samples.

## 3 Results

### 3.1 Clinical cases

Among 35 lymphoma samples included in this study, 22 samples were collected by endoscopic biopsy, 9 samples by fine-needle aspiration, and 4 samples by surgical resection. The median age of the cases was 11.1 (range: 3.9–13.6) years old. There were 19 males (12 were castrated) and 16 females (15 were spayed). The breeds of these dogs were Miniature Dachshunds (*n* = 8), Chihuahuas (*n* = 4), Toy Poodles (*n* = 4), Shibas (*n* = 4), Pomeranians (*n* = 2), Pugs (*n* = 2), Jack Russell Terriers (*n* = 2), French Bulldog (*n* = 2), and 1 each of English Cocker Spaniel, Miniature Schnauzer, Beagle, Norfolk Terrier, Pekingese, Papillon and mixed breed. By PARR analysis, clonal rearrangements of the *TCRγ* gene were shown in tumor samples of 24 dogs, the *IgH* gene in those from 2 dogs, and neither in those from 8 dogs. Amplification of the two control genes was verified to assess DNA quality for these 34 samples. In one dog (Dog 28), PARR could not be performed because of insufficient DNA quantity. Clinical information of the 35 dogs including age, sex, breed, and immunophenotype of tumor cells determined by PARR were shown in Supplementary Table S1.

### 3.2 WES

The samples of four dogs (Dogs 1–4) were subjected to WES analysis. Tumor samples were obtained by endoscopic biopsy in three dogs (Dogs 1–3) and by surgical resection in one dog (Dog 4). The lesions of all dogs were localized to the intestinal wall and adjacent lymph nodes. In WES, the mapping rate was more than 99% in each sample, and the average depths were 165× in tumors and 170× in peripheral blood. By comparison with the peripheral blood genome, 1910–4602 somatic mutations were called in each case (Supplementary Table S4). Among these called mutations, 9 mutations in Dog 1, 14 in Dog 2, 2 in Dog 3, and 147 in Dog 4 were extracted based on the filtering criteria described above (Table 1). The mutations in *TTBK1* and *NACC1* genes were commonly found in Dogs 1, 3, and 4, and Dogs 1 and 4, respectively (Table 2). The same mutation in *TTBK1* gene was observed in Dog 3 and 4. However, since the *TTBK1* mutation in Dogs 1, 3, and 4 was found to be located in the non-protein-coding region when verified on the CanFam3.1 database, the *TTBK1* was excluded from the following analysis. We also extracted the mutations in genes where mutations were commonly identified in human intestinal T-cell lymphoma, and we found the mutations in *ASXL3, SOCS3, PRDM1, FYN, TET2,* and *ZDBF2* genes in Dog 4 (Table 3) (9–11, 16, 17).

**Table 1:** The number of gene mutations extracted in each case in whole exome sequencing.

Putative impact on coding protein	Dog 1	Dog 2	Dog 3	Dog 4
High	4	6	0	76
Moderate	5	8	2	71
Total	9	14	2	147

**Table 2:** The gene mutations commonly observed among the cases in whole exome sequencing.

Gene	Chromosome	Mutation position	Reference sequence	Altered sequence	Annotation	Dogs that harbored the mutations
*TTBK1*	12	11774457	CCAGGCCCGGCCTGCGGGGCCCCCGCCCCGGGGCGCCCCG	C	Conservative inframe deletion	Dog 1
	12	11769826	A	AAAG	Conservative inframe insertion	Dog 3
	12	11769826	A	AAAG	Conservative inframe insertion	Dog 4
*NACC1*	20	49099680	C	CG	Frameshift	Dog 1
	20	49085421	GC	G	Frameshift	Dog 4

**Table 3:** The mutations identified by whole exome sequencing analysis in genes where mutations were commonly observed in human intestinal T-cell lymphoma.

Gene	Chromosome	Mutation position	Reference sequence	Altered sequence	Annotation	Dogs that harbored the mutations
*ASXL3*	7	56100053	C	T	Missense	Dog 4
*SOCS3*	9	2859773	CG	C	Frameshift	Dog 4
*PRDM1*	12	63489603	AC	A	Frameshift	Dog 4
*FYN*	12	68147532	C	T	Missense	Dog 4
*TET2*	32	26105744	C	T	Missense	Dog 4
*ZDBF2*	37	14892393	TA	T	Frameshift	Dog 4

### 3.3 Targeted NGS analysis

We decided to validate the 8 mutations in 7 genes that were extracted based on the results of WES. The *NACC1* mutation (c.204dupC), which was found in Dog 1 in WES analysis, could not be analyzed due to GC-rich sequences. Among the remaining 7 gene mutations observed in Dog 4 by WES analysis, those in *NACC1, ASXL3, SOCS3, PRDM1, FYN,* and *TET2* genes were not found in any of the 31 additional cases by targeted NGS analysis. As for the mutation in *ZDBF2* gene, the variant allele frequency was more than 0.15 in all tumor samples. Particularly high variant allele frequency was identified in tumor samples of the two dogs: 0.484 in Dog 4 and 0.394 in Dog 30. However, the same mutation was ubiquitously observed in all 17 peripheral blood samples with variant allele frequency ranging from 0.18 to 0.27.

Also, targeted NGS analysis that covered all exons revealed other point mutations in *ZDBF2* gene than that detected by WES: a missense mutation (c.485G > A) in Dog 17, two missense mutations (c.5339G > T, c.6731C > T) in Dog 33, and two missense mutations (c.5339G > T, c.7016C > T) in Dog 34 (Table 4).

**Table 4:** The mutations identified by Targeted Next-generation Sequencing analysis throughout the whole conding region of *ZDBF2* gene.

Gene	Chromosome	Mutation position (ROS_Cfam_1.0)	Mutation position (CamFam3.1)	Reference sequence	Altered sequence	Annotation	Variant allele frequency	Dogs that harbored the mutations
*ZDBF2*	37	14823603	14886987	G	A	Missense	0.465	Dog 17
	37	14828457	14891841	G	T	Missense	0.612, 0.472	Dog 33, 34
	37	14829849	14893233	C	T	Missense	0.607	Dog 33
	37	14830134	14893518	C	T	Missense	0.436	Dog 34

## 4 Discussion

In the present study using canine LCGIL, 8 gene mutation points in exon regions were identified by WES analysis. Among the 8 mutation points, 1 mutation could not be verified due to technical reasons, and 6 mutations were not observed by targeted NGS analysis using the additional LCGIL samples. In *ZDBF2* gene, 3 of 31 LCGIL cases possessed missense mutations in respective positions.

By WES analysis, 8 gene mutation points were identified in 7 genes where mutations were commonly identified in human intestinal T-cell lymphoma (9–11, 16, 17). *NACC1* regulates transcription related to cell proliferation, and *SOCS3* is a negative feedback regulator of the JAK–STAT pathway (18, 19). *FYN* encodes a tyrosine kinase which plays an important role in promoting T-cell activation (20). *ASXL3* and *PRDM1* are involved in histone modifications, and *TET2* in DNA demethylation (21–23). Although the function of the *ZDBF2* is unknown (24), it was possible that these mutations were associated with the pathophysiology of LCGIL in Dogs 1 and 4.

Then, 7 mutation points that were extracted by WES analysis were studied by targeted NGS analysis, and the variant allele frequency of the *ZDBF2* gene mutation was greater than 0.15 in all tumor samples and high variant allele frequency of the mutation in *ZDBF2* genes was identified in tumor samples of the two dogs. These results suggested the possibility that the *ZDBF2* mutation was present in all dogs with low frequencies, while higher frequencies were observed in some of the LCGIL tissues. It might be also possible that the mutation was caused by PCR error during amplification and sequence analysis considering that the mutation was located within 12 consecutive adenines. Also, the other 6 mutation points were not detected. The causes of the results might be that only the mutated positions identified by WES were investigated by targeted NGS analysis and other exon regions in six genes, which might harbor any mutations, were not analyzed in this study. In addition, targeted NGS analysis covering all exons of *ZDBF2* revealed additional point mutations in three LCGIL cases. *ZDBF2* is known as an imprinted gene that shows paternal allele-specific expression in humans and mice (24), and 44% of MEITL cases were shown to possess mutations in the *ZDBF2* gene (16). However, the biological implication of the *ZDBF2* gene mutation is unclear in tumors at present as described above, requiring further research.

Taken together, genes commonly mutated in most canine LCGIL cases were not found in the present study, indicating the possibility that canine LCGIL is a heterogeneous group of diseases with a variety of molecular aberrations in their background. Canine transmural gastrointestinal lymphoma was reported to be a complex disease with a wide range of histopathologic features and immunophenotypes (25). In addition, WES analysis of canine T-cell lymphoma that occurred in the lymph nodes showed that there was little overlap in the mutations among cases (6). It is also possible that non-genomic molecular aberrations may be critical for the development of canine LCGIL. A study explored microRNA expression patterns in canine intestinal T-cell lymphoma, and it showed differential expressions of several tumor-associated microRNAs compared to non-lymphoma tissues (26). Another study that analyzed genome-wide DNA methylation in dogs with gastrointestinal lymphoma reported hypermethylated sites that were common in most cases, suggesting the involvement of epigenetic abnormalities in tumorigenesis (27). Therefore, it might be needed to comprehensively analyze genomic and non-genomic molecular aberrations in each canine LCGIL case.

Limitations of the present study include that we could not confirm the immunophenotype of tumor cells by immunohistochemistry, although the results of PARR suggested that the tumor cells of most cases were derived from T-cells as previously reported (2). Also, mucosal lesions that were collected by endoscopy should contain non-neoplastic cells, including epithelial cells, plasma cells, and monocytic cells, and it was possible that the proportions of tumor cells in these endoscopically collected samples might be low. Indeed, the numbers of mutations found by WES was lower in the samples collected by endoscopic biopsy (Dogs 1–3) than that in a case where surgically resected tissue was used (Dog 4). Future studies using the tumor cells isolated from the tumor tissues are needed to investigate the gene mutations with higher sensitivity.

In conclusion, genes commonly mutated in most canine LCGIL cases were not found in the present study, and the results indicated that canine LCGIL may be a heterogeneous group of diseases with a variety of genetic aberrations. Future comprehensive analysis of genomic and non-genomic molecular aberrations is needed to be performed using the tumor cells isolated from the tumor tissues.

## Data Availability

Datasets obtained through WES analyses are available at the DDBJ Sequenced Read Archive repository with accession number PRJDB15563 (https://ddbj.nig.ac.jp/search/entry/bioproject/PRJDB15563).
