# Supplementary material for: Comprehensive investigation of gene mutations in canine large cell gastrointestinal lymphoma
Source: Front Vet Sci. 2025 Feb 19;12:1535446. doi: 10.3389/fvets.2025.1535446 (PMC11880205; doi:10.3389/fvets.2025.1535446)
Supplement: Supplementary file 1 [file Table_1.docx]

Supplementary Material

# Supplementary Data

# Supplementary Table 1. Signalment and sample information of dogs included in this study.

#

# Supplementary Table 2. Primer sequences used for targeted next-generation sequence analysis in this study.

#
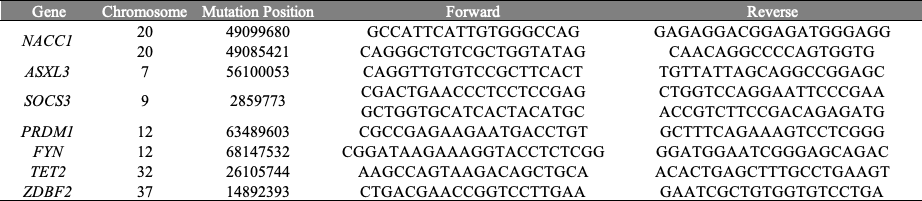


**Supplementary Table 3.** Primer sequences used for targeted next-generation sequence analysis throughout the whole exon region of *ZDBF2* gene.

**Supplementary Table S4.** The number of somatic mutations called by comparisons of tumor genome with the peripheral blood genome in each case.
